# Is Age-Related Macular Degeneration Associated with Stroke Among Elderly Americans?§

**DOI:** 10.2174/1874364100802010037

**Published:** 2008-03-08

**Authors:** Duanping Liao, Jingping Mo, Yinkang Duan, Ronald Klein, Ingrid U Scott, Kui A Huang, Haibo Zhou

**Affiliations:** 1 Department of Public Health Sciences, Pennsylvania State University College of Medicine, Hershey, PA, USA; 2 Pfizer Inc, New York, NY, USA; 3 University of Wisconsin-Madison, Madison, WI, USA; 4 Department of Biostatistics, University of North Carolina at Chapel Hill, Chapel Hill, NC, USA

**Keywords:** Age-related macular degeneration, stroke, Medicare medical claims, follow-up study.

## Abstract

**Objective:**

To investigate whether age-related macular degeneration (AMD) is associated with the development of ischemic and hemorrhagic stroke among elderly Americans.

**Design:**

Population-based cohort study.

**Participants:**

The five percent random sample of 2000-2003 Medicare enrollees was obtained. The cohort (n=1,519,086) consisted of enrollees who were aged 65 or older at the first two-year (January 1, 2000 to December 31, 2001).

**Methods:**

Baseline demographic variables and chronic conditions (AMD and type, history of myocardial infarction (MI), stroke, hypertension, and diabetes) were defined based on the occurrence of relevant ICD-9 codes in relevant diagnosis fields of the baseline Medicare Data. We excluded 215,900 persons who had a diagnosis of MI or stroke during baseline period to form a cohort of 1,303,186 individuals who were free of major cardio-cerebral vascular disease (CVD) at baseline.

**Main Outcome Measures:**

In two years of follow-up (January 1, 2002 to December 31, 2003), a total of 89,501 incident stroke cases were identified, including 80,018 ischemic, 7048 hemorrhagic, and 2,435 stroke cases of both types.

**Results:**

Baseline mean age was 75 years (Standard Divination=7.7), with 60% women and 88% whites. The prevalence of AMD was 10.6%, with 19.7% being neovascular AMD and 80.3% being non-neovascular AMD. Baseline age, gender, race, hypertension, and diabetes adjusted 2-year incident odds ratios and 95% confidence internal of stroke associated with AMD were 1.31 (1.26, 1.36) for neovascular AMD, 1.18 (1.15, 1.21) for non-neovascular AMD, and 1.21 (1.18, 1.23) for either neovascular or non-neovascular AMD.

**Conclusion:**

The findings are suggestive of an association between AMD, especially neovascular AMD, and incident stroke, independent of demographic factors and co-morbidity. These findings, if confirmed by other studies that control for smoking and other lifestyle covariables not measured in this study, suggest the possibility of shared common antecedents between stroke and AMD.

Age-related macular degeneration (AMD) is the leading cause of severe vision loss among elderly persons. However, the etiology of AMD is poorly understood. Genetic factors [1-3], smoking [4,5], and less consistently oxidative stress, long-term exposure to sunlight, or low levels of antioxidants [6,7], nutritional factors [8], obesity [9,10], and lipid levels [11,12] have been implicated as possible risk factors for the development of AMD. Many of these factors are also traditional risk factors for cardiovascular and cerebral vascular diseases, the leading cause of death and disability in the United States and other developed countries. The objective of this study is to investigate whether AMD, especially neovascular AMD, is related to the occurrence of incident stroke among elderly Americans.

## POPULATION AND METHODS

### Study Population

A 5% random sample of Medicare beneficiaries’ data and their medical reimbursement claims data were obtained from the Center for Medicare and Medicaid Services (CMS) after the CMS Privacy Board review and approval of the data request. We included all enrollees from this random sample during the first two years (January 1, 2000 to December 31, 2001) to form a cohort of persons aged >65 years (n=1,519,086), after excluding persons enrolled in Medicare before 65 years of age and persons with conflicting demographic information (date of birth, race, or gender) in the CMS demographic databases from different years. The research identifiable Carrier files, inpatient and outpatient files, and enrollment demographic files for the entire study period (2000-2003) were obtained from CMS and used for this study.

### Definitions of Key Variables

Baseline demographic variables and chronic conditions (AMD, history of myocardial infarction (MI), stroke, hypertension, and diabetes) were extracted from the Medicare Claims database during the 2-year baseline period, based on any occurrence of relevant ICD-9 codes in either the “Claim Diagnosis Code” or the “Line Diagnosis Code” field of the Medicare claim form. The definitions and their corresponding ICD-9 codes of AMD, MI, stroke, hypertension, and diabetes are summarized in Table **1**. The following algorithms and the ICD-9 codes were used to define stroke [13]:

- Any occurrence of 430, 431, 432.0-432.9, 433.01, 433.21, 433.31, 433.81 or 433.91, 434.00-434.91, or 436, on Medicare claim’s Diagnosis Code fields; or
- Any co-occurrences of V57 together with 342.00-342.92, 430, 431, 432.0-432.9, 433.00-433.91, 434.00-434.91, 435.0-435.9, 436, 437.0-437.9, 438.0-438.9, or 438.10-438.89 on Medicare claims’ diagnosis code fields; or
- Any co-occurrences of 433.00-433.91 or 435.0-435.9 together with 342.00-342.92, 430, 431, 432.0-432.9, 434.00-434.91, or 436 on Medicare claims’ diagnosis code fields.

A person was identified as having history of stroke if he/she met any of the above criteria during the baseline period. A person was identified as having a history of MI if there was any occurrence of ICD-9 codes 410.00 - 410.92. To derive a baseline cardio-cerebral vascular disease (CVD) free follow-up cohort, we excluded 215,900 individuals who had a diagnosis of stroke or MI during the baseline period. Thus, the sample for the prospective study of AMD and incident stroke included 1,303,186 individuals aged 65 years or older and free of CVD at baseline.

We used the Medicare claim’s data of the follow-up period (January 1, 2002 to December 31, 2003) to identify the first occurrence of incident stroke among the baseline CVD-free cohort members. A person was identified as having incident stroke if he/she met any of the above stroke criteria during the follow-up period. Hemorrhagic stroke is defined as the occurrence of ICD-9 codes 430 - 432 or 342, and ischemic stroke is defined as the occurrence of ICD-9 codes **(**433 - 438). We only analyzed the first occurrence of stroke in this study.

### Analysis Methods

The mean and standard deviation (SD) or proportion of demographic variables and co-morbidity were calculated. Logistic regression models were used to estimate the multivariable adjusted, as well as age group stratified, incident odds ratios and 95% confidence interval (CI) of stroke associated with baseline AMD. All analyses were performed using SAS software v9.13 (The SAS Institute, Cary, NC).

## RESULTS

In the 5% random sample of Medicare Beneficiaries, we identified 1,519,086 individuals, aged 65 or older at baseline, as our potential study population. After excluding 215,900 individuals who were identified as having history of stroke or MI during the two-year baseline period (January 1, 2000 to December 31, 2001), we included 1,303,186 individuals as our cohort members for the prospective study of the relationship between baseline AMD and incident stroke. During the two-year follow-up period (January 1, 2002 to December 31, 2003), we identified 89,501 individuals as having incident stroke, with a two-year cumulative incidence of 6.9%. Among them, 80,018 (89.4%) were classified as ischemic stroke, 7048 (7.9%) as hemorrhagic stroke, and 2,435 (2.7%) as having stroke diagnostic codes for both types of stroke. In this cohort, 10.6% individual had AMD at baseline. 19.7% of these persons had neovascular AMD and 80.3% had non-neovascular AMD. The descriptive characteristics of the study population, stratified by baseline AMD type, are summarized in Table **2**. Briefly, in the cohort, the average age was 75 years (SD=7.7), with 60% women and 88% whites. Among persons without AMD (non-AMD group), the average age was 75 (SD=7.5) years, with 59% women and 87% whites, and the prevalence of hypertension and diabetes was 61% and 23%, respectively. At the baseline, the AMD group was older with higher proportions of women and whites, and had higher prevalence of hypertension and diabetes than the non-AMD group (all p<0.01). As presented in Table **2**, in the univariate analysis without adjusting for any potential confounders, the incidence of stroke, both ischemic and hemorrhagic forms, was higher among persons with AMD than among persons without AMD. Table **3** shows the age, race, gender, hypertension, and diabetes adjusted two-year cumulative incidences (95% CI) of stroke according to baseline AMD status and age group. Consistently across all age strata, the incidence of stroke was higher among persons with baseline AMD for both neovascular and non-neovascular AMD, and the incidence of stroke was the highest in persons with neovascular AMD and older age group. Table **4** presents the age, gender, race, hypertension, and diabetes adjusted two-year incident odds ratios and 95% CI of all stroke, ischemic stroke, and hemorrhagic stroke associated with AMD, in the entire cohort and stratified by age group. Similar to the patterns observed in Table **3**, persons with AMD were more likely to develop stroke when compared with persons without AMD at baseline (referent group). For example, in the all-age group, persons with neovascular AMD at baseline had a 31% higher risk of developing stroke within two years than persons without AMD at baseline. When stratified by stroke types, persons with AMD at baseline, either neovascular or non-neovascular AMD, had approximately 15-30% higher risk of developing ischemic or hemorrhagic stroke within two years of follow-up, and the association was not different by stroke types. Overall, the data on Table **4** are indicative of slightly higher risk of stroke associated with neovascular AMD than non-neovascular AMD, and the AMD and stroke association was similar in magnitude for both types of stroke.

It should be noted that in the results presented in Table **4**, we included all first incident stroke cases (n=89,501) for the analysis of all stroke. However, when stratified by stroke types, we only included solid ischemic stroke (n=80,018) and solid hemorrhagic stroke (n=7,048), by excluding 2,435 stroke cases who had stroke diagnostic codes for both types of stroke in their first stroke claim forms. We performed additional sensitivity analysis by classifying these “mixed” cases either as ischemic or hemorrhagic stroke, and the pattern of associations (data not shown) did not change meaningfully from that presented in Table **4**.

## DISCUSSION

Age-related macular degeneration is highly prevalent among elderly individuals in developed countries. An earlier study (1980) estimated that 6% of the US population aged 65-74 years, and 20% of those older than 75 years, are affected by AMD [14], which causes 54% of legal blindness in persons 65 years of age or older [14]. It was recently estimated that 1.56 million US citizens 65 years of age and older are affected by neovascular AMD, and this number is estimated to increase to almost 3 million by 2020 [15]. Data from one study suggested that the 5-year incidence of neovascular AMD, which has been reported to be responsible for 90% of severe vision loss associated with AMD, in Medicare Beneficiaries was 18.7 per 1000 [16].

Several epidemiological studies have provided data concerning risk factors associated with AMD. The most prominent risk factors are older age, genetic factors [1-3], cigarette smoking [4, 5], and less consistently oxidative stress, long-term exposure to light, low levels of antioxidants [6,7], and other nutritional factors [8], obesity [9, 10], and serum lipid levels [11, 12]. However, the pathological processes through which these factors may contribute to AMD remain poorly understood. Others have reported that women and whites are at higher risk of AMD [14, 16]. The associations between AMD and hypertension and diabetes mellitus have been found to be inconsistent [17-29].

In a previous study using the same data as this study, we found that AMD was associated with older age, female gender, and white race. Furthermore, AMD, especially neovascular AMD, was cross-sectionally associated with the presence of hypertension, diabetes mellitus, and MI, independent of age, gender, and race[30]. In the prospective analysis, we reported that baseline AMD was significantly associated with the development of incident MI [30].

In the current study, the results stratified by AMD-type and stroke-type, show that both neovascular AMD and non-neovascular AMD are associated with incident ischemic and hemorrhagic stroke. However, neovascular AMD is a stronger predictor than non-neovascular AMD, and the AMD is a stronger predictor for ischemic than for hemorragic stroke. For example, compared to non-AMD group, baseline neovascular AMD was associated with a 31% increased risk of incident ischemic stroke, *vs* 18% for non-neovascular AMD. Similarly, baseline neovascular AMD was associated with a 26% increased risk of incident hemorrhagic stroke, *vs* 22% for non-neovascular AMD. To our knowledge, this is the largest population-based study to demonstrate a significant prospective association between AMD and stroke. The results from this study are similar to that reported by Wong and colleagues [31] in the population-based Arthrosclerosis Risk in Communities (ARIC) study - after adjusting for age, sex, ethnicity, and site, blood pressure, diabetes, cigarette smoking, and use of antihypertensive medications, the presence of early-stage AMD was associated with a 85% increased risk of incident stroke as compared to persons without AMD. These data suggest that AMD and stroke may share some common antecedents. We speculate that the presence of AMD is a risk indicator of higher levels of various exposures not measured in the current study, e.g., cigarette smoking, inflammation [32] and subclinical artherosclerosis [22, 33]. Although not adjusted for these “not measured” confounding factors, these weak associations (all odds ratios less then 2.00) suggest that AMD is a “minor” predictor of incident stroke under an etiological model. However, a public health perspective argues that estimates of the magnitude observed in this study would suggest that the population burden of stroke associated with AMD is still high due to high prevalence of AMD among elderly Americans.

Medicare claims data from Medicare beneficiaries have been used for various research projects. The enrollment rate in the Medicare system among the elderly American population remains extremely high. For example, there are currently about 40 million Americans aged 65 years or older enrolled in Medicare, which accounts for approximately 98% of elderly Americans. Thus, medical claims data from these Medicare beneficiaries provide a very large sample size, which would be unmatchable by any other population-based studies. Additionally, Medicare data provide the greatest representative sample of the US population aged 65 years or older.

There are limitations of using Medicare claims data. First, the claims based data vary in terms of sensitivity and specificity for the diagnosis of chronic conditions. For example, in a study comparing the Medicare claim-based ICD-9 codes with hospital chart review, it was shown that the sensitivity and specificity for the claim-based diagnosis of hypertension were 0.61 and 0.95, respectively [34]. In the same study, the sensitivity and specificity for the claim-based diagnosis were 0.75 and 0.99 for diabetes, 0.57 and 0.96 for coronary heart diseases, and 0.35 and 0.99 for stroke [34]. In another similar study, the sensitivity and specificity were 0.69 and 0.83 for hypertension, 0.90 and 0.93 for diabetes, 0.58 and 0.93 for heart failure, and 0.68 and 0.95 for glaucoma [35]. Similarly, our classifications of AMD and incident stroke, the key predictor and outcome variables, were solely based on the claims diagnostic codes, without the ability to obtain results from medical charts pertaining to eye examinations, retinal photographs, cerebral CAT scan or MRI, or any other neurological tests. Therefore, we cannot confirm the diagnosis of these conditions in this study, nor can we provide direct assessment of the sensitivity and specificity of the major variables analyzed in this study. However, our findings would have been biased toward the null if the misclassification due to limited sensitivity, and to a less degree limited specificity, were non-differential, that is, there was no difference in misclassifying incident stroke due to AMD status. In addition, in this report, ICD-9 code 362.53 was used in the algorithm to identify persons as neovascular AMD (to be consistent with the report by Lee and co-workers [36]). In practice, this code is used to code other non-AMD conditions also. We performed two additional sensitivity analyses by either classifying ICD-9 code 362.53 as non-neovascular, or as non-AMD, and the pattern of association did not change meaningfully (data not shown). Second, because the data are claim based, we did not have the ability to ascertain the chronic conditions prior to the study period. Therefore, we can only analyze the chronic conditions within the study period and assume that the absence of a condition during the baseline period represents a negative history of such condition. For example, when we excluded persons with stroke at baseline period to derive a cohort of baseline stroke-free cohort, we may have misclassified some individuals as “stroke-free” because we assumed they did not have a history of stroke prior to the year 2000. However, it is possible that some individuals in this cohort had a stroke prior to year 2000 but did not seek medical services for their past stroke during the two-year baseline period. As a result, he/she would be classified as a stroke-free individual at baseline. Thirdly, we cannot ascertain that a claim for stroke diagnosis during the follow-up period truly represents an incident stroke during that period. It is possible that the Medicare service was provided for a stroke that occurred prior to the follow-up or even before year 2000. Another major limitation of this study is the unavailability of established stroke risk factors from the claims databases. For example, data on cigarette smoking (both history and intensity), obesity, physical activity, and alcohol consumption cannot be derived from the CMS database. Therefore, we cannot control for the potential confounding by these risk factors, nor can we assess the direct impact of such potential confounding on our findings. However, based on the population based Atherosclerosis Risk in Communities (ARIC) study data [31], which reported a significant association between AMD and incident stroke after statistically controlling for smoking status and other lifestyle factors, it is not likely that the associations observed in our study can be fully attributable to the lack of control of these lifestyle factors.

Despite the inherent limitations of using Medicare claims data as outlined above, the validity of these data is supported by the fact that our findings of AMD being associated with older age, female gender, white race, hypertension, and diabetes are consistent with the findings in several other studies using different populations and methods. Our prospective findings indicate that baseline AMD, especially neovascular AMD, is associated with higher risks of ischemic and hemorrhagic incident strokes, independent of baseline demographic factors and co-morbidity. These findings, if confirmed by other studies that control for smoking and other lifestyle covariables not measured in this study, are suggestive of the possibility of shared common antecedents between AMD and stroke.

## Figures and Tables

**Table 1.** Definition of Key Variables and their Corresponding ICD-9 Codes

Condition	ICD-9 Code	Definition
Stroke [13]	342 430 431 432 433 434 435 436 437 438 V57	Hemiplegia and hemiparesis Subarachnoid hemorrhage Intracerebral hemorrhage Other and unspecific intracranial hemorrhage Occlusion and stenosis of precerebral arteries Occlusion of cerebral arteries Transient cerebral ischemia Acute, but ill-defined cerebovascular disease Other ill-defined cerebrovascular disease Late effects of cerebrovascular disease Care involving use of rehabilitation procedures
Neovascular AMD [16,35]	362.42-362.43 362.52 362.53	Serous or hemorrhagic detachment of retinal pigment epithelium Exudative senile macular degeneration of retina Cystoid macular degeneration of retina
Non-neovascular [16, 35]	362.5 362.50 362.51 362.57	Degeneration of macula and posterior pole Unspecified macular degeneration (senile) of retina Nonexudative senile macular degeneration of retina Drusen (degenerative) of retina
Hypertension	401.0 - 401.9 402.00 - 402.91 403.00 - 403.91 404.00-404.93	Essential hypertension Hypertensive heart disease Hypertensive renal disease Hypertensive heart and renal disease
Diabetes	250.00-250.93 357.2 362.01 362.02 366.41	Diabetes Polyneuropathy in diabetes Background diabetic retinopathy Proliferative diabetic retinopathy Diabetic cataract
MI	410.00 - 410.92	Acute myocardial infarction

AMD: Age-related Macular Degeneration; MI: Myocardial Infarction.

**Table 2.** Characteristics (Mean, SD, or Proportion) of Study Population by Baseline AMD Status

	Non-AMD (N=1,165,348)	Neovascular AMD (N=27,193)	Non-neovascular AMD (N=110,645)
Age (years)	75 (7.5)	80 (7.2)	80 (7.5)
Female (%)	59	65	68
White (%)	87	94	94
Hypertension*	61	74	71
Diabetes*	23	29	24
Incident Stroke (%)	6.6	10.3	9.2

* Diagnostic codes submitted during the 2-year baseline period.

AMD: Age-related Macular Degeneration; SD: Standard Deviation.

**Table 3.** Multivariable Adjusted* 2-Year Incidence (%) and 95% CI of Stroke, Stratified by AMD and Age Group

	All Age Group N=1,303,186	Age Group (Year)			
		65-69 (N=345,309)	70-74 (N=315,770)	75-79 (N=272,825)	≥ 80 (N=369,282)
Non-AMD	6.35 (6.30, 6.40)	4.05 (3.98, 4.12)	5.29 (5.20, 5.37)	7.03 (6.92, 7.13)	9.26 (9.16, 9.37)
Non-neovascular AMD	7.41 (7.27, 7.56)	4.25 (3.89, 4.64)	5.94 (5.62, 6.28)	8.12 (7.80, 8.45)	10.78 (10.52,11.04)
Neovascular AMD	8.15 (7.85, 8.45)	5.77 (4.98, 6.68)	6.25 (5.61, 6.95)	8.44 (7.81, 9.11)	11.73 (11.21,12.28)

* Adjusted for age within each age group, gender, race, hypertension, and diabetes.

CI: Confidence Interval; AMD: Age-related Macular Degeneration.

**Table 4.** Multivariable Adjusted* 2-Year Incident Odds Ratios and 95% CI of Stroke, Stratified by AMD-Type and Age Group

	All Age Group	Age Group			
		65-69	70-74	75-79	≥ 80
***Combined Stroke (n=89,501)***					
Non-AMD	1.00	1.00	1.00	1.00	1.00
All AMD	1.21 (1.18, 1.23)	1.15 (1.06, 1.24)	1.16 (1.10, 1.22)	1.20 (1.15, 1.25)	1.20 (1.17, 1.24)
Non-neovascular AMD	1.18 (1.15, 1.21)	1.06 (0.96, 1.16)	1.14 (1.08, 1.22)	1.19 (1.13, 1.24)	1.18 (1.15, 1.22)
Neovascular AMD	1.31 (1.26, 1.36)	1.50 (1.28, 1.75)	1.22 (1.09, 1.36)	1.24 (1.14, 1.35)	1.30 (1.23, 1.37)
***Ischemic Stroke (n=80,018)***					
All AMD	1.20 (1.18, 1.23)	1.14 (1.05, 1.24)	1.15 (1.09, 1.22)	1.21 (1.16, 1.26)	1.20 (1.16, 1.23)
Non-neovascular AMD	1.18 (1.15, 1.20)	1.05 (0.95, 1.16)	1.14 (1.07, 1.21)	1.20 (1.14, 1.26)	1.17 (1.14, 1.21)
Neovascular AMD	1.31 (1.26, 1.37)	1.49 (1.26, 1.75)	1.22 (1.08, 1.38)	1.25 (1.14, 1.37)	1.29 (1.22, 1.37)
***Hemorrhagic Stroke (n=7,048)***					
All AMD	1.23 (1.15, 1.32)	1.26 (0.94, 1.63)	1.20 (0.99, 1.44)	1.17 (1.01, 1.35)	1.23 (1.13, 1.35)
Non-neovascular AMD	1.22 (1.14, 1.32)	1.19 (0.86, 1.60)	1.15 (0.93, 1.42)	1.17 (1.00, 1.37)	1.24 (1.12, 1.36)
Neovascular AMD	1.26 (1.10, 1.45)	1.53 (0.84, 2.54)	1.38 (0.92, 1.98)	1.16 (0.85, 1.55)	1.21 (1.00, 1.45)

* Adjusted for age within each age group, gender, race, hypertension, and diabetes. Persons without AMD during the baseline period comprised the referent group.

CI: Confidence Interval; AMD: Age-related Macular Degeneration.
